# Praktische Empfehlungen zum Screening und Management von Funktionsstörungen der Nebennierenrinde bei einer akuten SARS-CoV-2-Infektion

**DOI:** 10.1007/s00108-021-01236-1

**Published:** 2021-12-20

**Authors:** Jimmy Masjkur, Andreas Barthel, Waldemar Kanczkowski, Gregor Müller, Stefan R. Bornstein

**Affiliations:** 1 Medizinische Klinik und Poliklinik III, Universitätsklinikum Carl Gustav Carus Dresden, Fetscherstraße 74, 01307 Dresden, Deutschland; 2 Else Kröner-Fresenius-Stiftung (EKFS) Clinician Scientist-Programm, UniversitätsCentrum für Seltene Erkrankungen (USE) am Universitätsklinikum Dresden, Dresden, Deutschland; 3 Medicover Bochum MVZ, Bochum, Deutschland

**Keywords:** Morbus Addison, Cushing-Syndrom, „Coronavirus disease 2019“ (COVID-19), Sekundäre Nebennierenrindenfunktionsstörungen, Glukokortikoide, Addison disease, Cushing syndrome, Coronavirus disease 2019 (COVID-19), Adrenal cortex insufficiency, secondary, Glucocorticoids

## Abstract

Erkrankungen der Nebennierenrinde erfordern im Rahmen der Severe-acute-respiratory-syndrome-coronavirus-2(SARS-CoV-2)-Pandemie eine besondere Aufmerksamkeit. Zum einen können SARS-CoV-2-Infektionen sich auch extrapulmonal manifestieren und endokrine Störungen – insbesondere im Bereich der Nebennierenrinde – verursachen. Zum anderen sind Patienten mit einer vorbestehenden Nebennierenrindeninsuffizienz oder einem Hyperkortisolismus durch eine schwerwiegende Infektion wie etwa mit SARS-CoV‑2 besonders gefährdet, zusätzliche Komplikationen oder einen schwerwiegenderen Verlauf einer akuten SARS-CoV-2-Infektion mit erhöhter Mortalität zu erleiden. Insbesondere bei hämodynamisch instabilen Patienten mit SARS-CoV-2-Infektion müssen deshalb auch Erkrankungen der Nebennieren differenzialdiagnostisch erwogen und gegebenenfalls abgeklärt werden, falls diese nicht bereits anamnestisch bekannt sind. Weiterhin kann auch die Therapie einer SARS-CoV-2-Infektion mit hohen Glukokortikoiddosen über einen längeren Zeitraum eine sekundäre Nebennierenrindeninsuffizienz verursachen. Wir stellen hier deshalb eine Praxisempfehlung zur Erkennung und Therapie von Nebennierenfunktionsstörungen bei Patient*innen mit SARS-CoV-2-Infektion vor.

## Abstract (en)

Diseases of the adrenal cortex require particular attention during the severe acute respiratory syndrome coronavirus 2 (SARS-CoV-2) pandemic. Firstly, SARS-CoV‑2 infections can give rise to extrapulmonary manifestations and cause endocrine disorders, particularly in the adrenal cortex. Furthermore, patients with pre-existing insufficiency of the adrenal cortex or hypercortisonism are particularly at risk from a severe infection such as SARS-CoV‑2, to suffer from additional complications or a more severe course of a SARS-CoV‑2 infection with a higher mortality. Especially in hemodynamically unstable patients with a SARS-CoV‑2 infection, diseases of the adrenal glands should also be considered in the differential diagnostics and if necessary clarified, if this is not already known. Prolonged treatment of patients with a SARS-CoV‑2 infection with regimens containing high doses of glucocorticoids can also result in a secondary adrenal insufficiency. In order to address these special aspects, some practical recommendations for the diagnostic and therapeutic management of functional disorders of the adrenal glands in patients with a SARS-CoV‑2 infection are therefore presented.

Das Auftreten von Funktionsstörungen der Nebennierenrinde wurde in Fallberichten bei Patient*innen mit Severe-acute-respiratory-syndrome-coronavirus-2(SARS-CoV-2)-Infektion beschrieben [1–3]. In der Tat wurde SARS-CoV‑2 in den Nebennierenzellen einiger Patient*innen mit schwerem Verlauf einer SARS-CoV-2-Infektion nachgewiesen, was darauf hindeutet, dass dieses Organ ebenfalls zu den Zielorganen der Infektion gehört [4]. In der vorliegenden Übersicht werden die direkten potenziellen Auswirkungen einer SARS-CoV-2-Infektion auf die Funktion der Nebennierenrinde sowie die Auswirkungen einer solchen Infektion auf den Krankheitsverlauf bei Patient*innen mit einer vorbestehenden Nebennierenerkrankung diskutiert. Darüber hinaus fassen wir die diagnostischen und therapeutischen Vorgehensweisen anhand praktischer Empfehlungen zusammen.

## Primäre Nebennierenrindeninsuffizienz

Eine primäre Nebennierenrindeninsuffizienz („primary adrenal insufficiency“ [PAI]) ist durch eine Funktionseinschränkung der Nebennieren bis zum kompletten Funktionsverlust charakterisiert. Ursächlich zurückzuführen ist dieses Krankheitsbild häufig auf eine autoimmun bedingte Adrenalitis, seltener auch auf vielfältige anderweitige erworbene Ursachen wie

- Infektionen, beispielsweise
  - Tuberkulose,
  - Human-immunodeficiency-virus(HIV)-Infektion/„acquired immunodeficiency syndrome“ (AIDS),
  - Zytomegalievirus(CMV)-Infektion,
  - Candidiasis;
- tumoröse Infiltrationen;
- Sepsis;
- adrenale Hämorrhagien oder
- unerwünschte Medikamenteneffekte, beispielsweise durch
  - Ketoconazol,
  - Mitotan,
  - Pembrolizumab und
  - andere Checkpointinhibitoren.

Darüber hinaus gibt es auch eine Palette angeborener Ursachen wie das adrenogenitale Syndrom oder die Adrenoleukodystrophie. Patient*innen mit einer PAI haben nicht nur einen Mangel an Glukokortikoiden, sondern häufig auch einen Mineralokortikoidmangel. Bei Patient*innen mit PAI besteht ein erhöhtes Risiko, an einer SARS-CoV-2-Infektion zu erkranken und danach schwere Komplikationen zu entwickeln [1, 5]. Dies könnte im Rahmen einer gewissen möglicherweise bestehenden Immunkompromittierung bei diesen Patient*innen erklärt werden. Darüber hinaus lässt sich eine erhöhte Mortalität bei PAI-Patient*innen mit einer schweren Atemwegsinfektion im Rahmen einer SARS-CoV-2-Infektion auch daraus ableiten, dass diese Patient*innen aufgrund des Fehlens der endogenen adrenokortikotropen Regulation leichter hämodynamisch instabil werden und dekompensieren, weil die endogene Gluko- und Mineralokortikoidsekretion in einer solchen Situation therapeutisch nicht optimal abgebildet werden kann. Möglicherweise interferiert eine SARS-CoV-2-Infektion zudem bei ansonsten „gesunden“ Patient*innen mit der physiologischen adrenokortikotropen Regulation, denn viele infizierte Patient*innen zeigen eine auffällige hämodynamische Instabilität. So wurde beschrieben, dass bei 22–67 % der hospitalisierten Patient*innen mit SARS-CoV-2-Infektion eine Vasopressortherapie erforderlich wurde [1, 6].

Weiterhin ist zu bedenken, dass bei einer vasopressorresistenten Hypotonie im Rahmen einer SARS-CoV-2-Infektion auch eine vorbestehende PAI als Ursache übersehen werden kann und zumindest differenzialdiagnostisch erwogen werden sollte. Der Begriff „critical illness-related corticosteroid insufficiency“ (CIRCI) wird als eine in der klinischen Situation unzureichende Glukokortikoidsynthese, -sekretion und -wirkung definiert. Die CIRCI geht mit einem Anstieg der zirkulierenden biologischen Entzündungs- und Gerinnungsmarker einher, was häufig bei Patient*innen mit schwerer SARS-CoV-2-Infektion beobachtet wird [2, 7, 8].

SARS-CoV‑2 kann möglicherweise direkt die adrenokortikalen Zellen angreifen und schädigen

**Figure 1:**
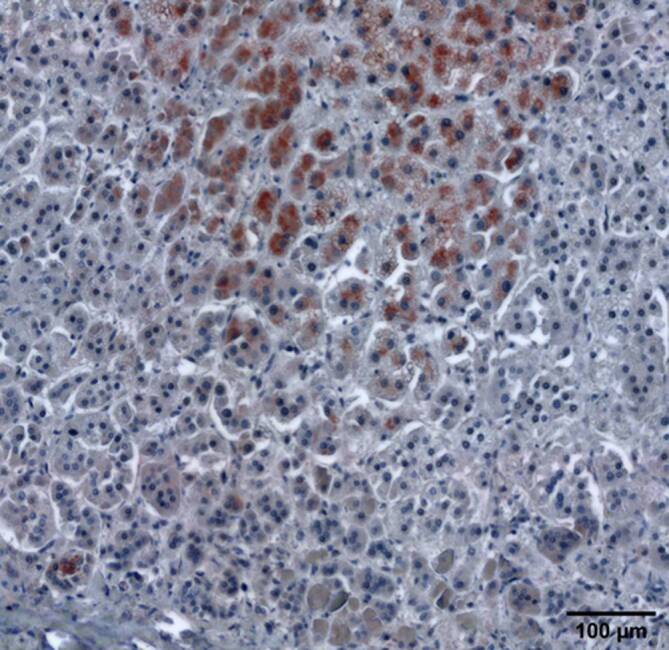

Das SARS-CoV-2-Virus kann über das virale Spike-Protein (S-Protein) durch mehrere Mechanismen direkt die Zellen der Nebenniere infizieren und infolgedessen eine Funktionsstörung der Nebennierenrinde auslösen, die zur Manifestation einer PAI führt. Das S‑Protein lagert sich an das „angiotensin-converting enzyme 2“ (ACE2) an und verwendet die zelluläre Serinprotease (TMPRSS2) für das S‑Protein-Priming in Lungenzellen. Auch in der Nebenniere wurde mit immunhistochemischen Methoden eine ACE2- sowie TMPRSS2-Expression nachgewiesen, zudem das S‑Protein von SARS-CoV‑2. Diese Ergebnisse deuten darauf hin, dass SARS-CoV‑2 möglicherweise direkt die adrenokortikalen Zellen angreifen und schädigen kann, was dann zu einer Störung der Glukokortikoid- und auch Mineralokortikoidsynthese sowie zu einer Manifestation einer PAI führen kann [1, 9, 10]. Abb. 1 zeigt die Expression von SARS-CoV-2-Spike-Protein in der Nebenniere von humanem Autopsiematerial. Bei Autopsien von Patient*innen, die an einer SARS-CoV-2-Infektion verstorben waren, konnten ebenfalls degenerative und nekrotische Veränderungen zusammen mit einer Infektion von Nebennierenzellen durch SARS-CoV‑2 nachgewiesen werden, was auf möglicherweise bestehende direkte zytopathische Effekte des Virus hindeutet.

Allerdings muss einschränkend bemerkt werden, dass auch anderweitige pathologische Effekte im Rahmen eines schweren durch SARS-CoV‑2 verursachten septischen Krankheitsbilds, beispielsweise venöse Thromboembolien oder Hämorrhagien im Bereich adrenaler Gefäße, eine PAI verursachen können. So fanden sich im Sektionsgut von Patient*innen, die an einer schweren SARS-CoV-2-Infektion verstorben waren, häufig auch akute fibrinoide Nekrosen kleiner Gefäße im Nebennierenparenchym, in der Kapsel und im periadrenalen Fettgewebe mit subendothelialer Vakuolisierung und apoptotischen Zelltrümmern ohne signifikante Anzeichen einer Entzündung, parenchymaler Infarkte oder Thrombosen, was auf einen möglicherweise bestehenden spezifischeren Effekt hindeuten könnte [3–5, 11–13].

## Hyperkortisolismus, adrenales Cushing-Syndrom und autonome Kortisolsekretion

Im Gegensatz zu einem iatrogenen Hyperkortisolismus, der häufig bei der Therapie chronisch-entzündlicher oder maligner Erkrankungen besteht, handelt es sich beim endogenen Cushing-Syndrom um eine seltene Störung, die durch eine Überproduktion von Kortisol verursacht wird. Diese ist in etwa 80 % der Fälle vom adrenokortikotropen Hormon (ACTH) abhängig. Im Gegensatz dazu ist der Hyperkortisolismus beim adrenalen Cushing-Syndrom (AC) ACTH-unabhängig. Bei einer autonomen Kortisolsekretion (ACS) handelt es sich nur um einen leichten ACTH-unabhängigen adrenalen Hyperkortisolismus aufgrund eines Nebennierenadenoms oder einer Nebennierenhyperplasie, die typischerweise zufällig im Rahmen einer Bildgebung zur Abklärung eines anderen Krankheitsbilds entdeckt wurde. Obwohl die Prävalenz einer ACS wesentlich höher ist als die des AC, ist eine klinische Diagnose aufgrund der fehlenden typischen Symptome des Hyperkortisolismus sehr unwahrscheinlich. Patient*innen mit ACS weisen allerdings häufig eine oder mehrere Komponenten des metabolischen Syndroms auf und haben damit ein erhöhtes kardiovaskuläres Risiko [14–16]. Dementsprechend ist auch ein milder chronischer Hyperkortisolismus durch entsprechende Veränderungen gekennzeichnet, etwa durch eine gewisse Immunkompromittierung sowie die Entwicklung von Übergewicht und Adipositas, Diabetes mellitus Typ 2, arterielle Hypertonie, Hyperkoagulabilität, Myopathie und neuropsychiatrische Störungen [15, 17].

Ein chronischer Hyperkortisolismus – ob endogen oder exogen – kann aufgrund der Immunkompromittierung zu einer erhöhten SARS-CoV-2-Infektions-Anfälligkeit führen. Aus diesem Grund wäre zu erwarten, dass Patient*innen mit einem Hyperkortisolismus auch ein erhöhtes Risiko für eine SARS-CoV-2-Infektion mit schwerem klinischem Verlauf haben. Es gibt allerdings nur wenige verfügbare Daten über Patient*innen mit Cushing-Syndrom, die von SARS-CoV‑2 infiziert wurden. Weitere Beobachtungen deuten darauf hin, dass die Mortalität bei SARS-CoV-2-Patient*innen mit Herzerkrankungen und metabolischem Syndrom im Vergleich zu anderen Patient*innen deutlich höher ist. Darüber hinaus wurde auch beschrieben, dass häufige hyperkortisolismusassoziierte laborchemische Veränderungen – nämlich Lymphopenie oder Leukozytose, erhöhte D‑Dimere und eine verlängerte Prothrombinzeit – mit einem erhöhten SARS-CoV-2-bedingten Sterberisiko assoziiert sind. Es ist unklar, ob Patient*innen mit endogenem Hyperkortisolismus oder unter einer Glukokortikoidtherapie eine unzureichende Immunantwort auf SARS-CoV-2-mRNA-Impfstoffe haben. Allerdings ist zu vermuten, dass bei einem Hyperkortisolismus die Wirksamkeit der Impfstoffe durch die immunsuppressive Wirkung vermindert ist [17–19]. Unter diesen Gesichtspunkten erscheint es plausibel anzunehmen, dass der klinische Verlauf einer SARS-CoV-2-Infektion vom Schweregrad eines Hyperkortisolismus abhängig ist. Bei Patient*innen mit ausgeprägtem Hyperkortisolismus wäre somit davon auszugehen, dass im Falle einer SARS-CoV-2-Infektion auch bei fehlenden oder geringen klinischen Symptomen und unauffälligen Entzündungsparametern in der akuten Phase eine intensivmedizinische Versorgung rascher erforderlich wird [17, 20, 21].

## Praktische Empfehlungen

Schwerwiegende und seltene Erkrankungen der Nebennieren wie PAI, AC und ACS sollten bei Patient*innen mit SARS-CoV-2-Infektion differenzialdiagnostisch in Betracht gezogen werden, insbesondere bei Eintreten einer hämodynamischen Instabilität. Bei solchen Patient*innen sollte eine zusätzliche diagnostische Abklärung durchgeführt werden, auch wenn aus der Vorgeschichte keine Nebennierenerkrankung bekannt ist. Um hier effizient vorzugehen, schlagen wir für die praktische Diagnostik und Therapie folgendes Vorgehen vor.

### Diagnostik

Patient*innen mit einer SARS-CoV-2-Infektion sollten zunächst auf der Grundlage der von der Weltgesundheitsorganisation (WHO) festgelegten Leitlinie [22] nach klinischen Gesichtspunkten klassifiziert werden. Anhand dieser Klassifizierung werden alle Patient*innen in drei Kategorien eingeteilt [23]:

- Gruppe I: ambulante Gruppe, definiert als ambulante Patient*innen mit bestätigter SARS-CoV-2-Infektion und ohne Indikation für einen Krankenhausaufenthalt
- Gruppe II: Gruppe mit leichter Erkrankungssymptomatik, definiert als hospitalisierte Patient*innen mit bestätigter SARS-CoV-2-Infektion, die keine Beatmungsunterstützung benötigen
- Gruppe III: Gruppe mit schwerer Erkrankung, definiert als hospitalisierte Patient*innen mit bestätigter SARS-CoV-2-Infektion, die Beatmungsunterstützung benötigen

Unabhängig von der klinischen Symptomatik erfolgt das Screening auf Funktionsstörungen der Nebennierenrinde nur im stationären Bereich (Gruppen II und III). Hier werden eine basale Probe für ACTH und Kortisol sowie eine Bestimmung der Serumelektrolyte als Erstlinientest zu Beginn einer SARS-CoV-2-Infektion empfohlen, um schnell einen Hypokortisolismus auszuschließen. Die Bestimmung des Speichelkortisols sollte wegen des Risikos einer SARS-CoV-2-Infektion vermieden werden. Ein über das Zweifache der oberen Normgrenze erhöhter Plasma-ACTH-Wert bei Patient*innen mit bestätigtem Kortisolmangel spricht für eine PAI. Hyponatriämie und Hyperkaliämie würden diese Diagnose ebenfalls unterstützen, auch wenn sie in der akuten Phase nicht immer vorhanden sind.

Für die Diagnose einer primären Nebennierenrindeninsuffizienz gilt der ACTH-Test als Goldstandard

**Figure 2:**
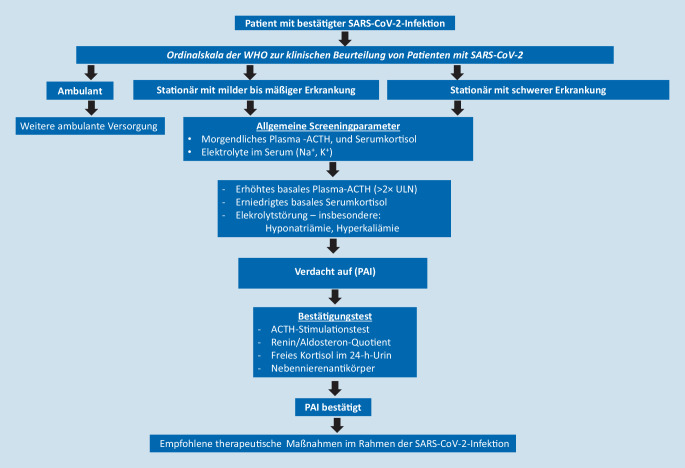

Für die Diagnose einer primären Nebennierenrindeninsuffizienz gilt der ACTH-Test (Synacthen[Corticotropin]-Test) als Goldstandard. Hier erfolgt nach Applikation einer Standarddosis Synacthen (250 µg für Erwachsene und Kinder > 2 Jahre, 15 µg/kg für Säuglinge bzw. 125 µg für Kinder < 2 Jahre) eine Bestimmung der Serumkortisolkonzentration. Kortisolspitzenwerte unter 500 nmol/l (18 µg/dl) nach 30 oder 60 min in Kombination mit einem erhöhten ACTH-Spiegel sind diagnostisch für eine PAI. Bei Vorliegen einer PAI wird die Bestimmung von Plasmarenin und Aldosteron zur Diagnose eines potenziellen Mineralokortikoidmangels empfohlen. Bei neu bestätigter Nebennierenrindeninsuffizienz sollte im nächsten Schritt die Ätiologie differenzialdiagnostisch abgeklärt werden, wobei in der Regel umfassendere endokrinologische Expertise erforderlich ist [24–26]. Da die schnelle Erkennung einer primären Nebennierenrindeninsuffizienz bei Patient*innen mit SARS-CoV-2-Infektion lebensrettend sein kann, ist in Abb. 2 ein diagnostischer Algorithmus dargestellt. Bei einer sekundären Nebennierenrindeninsuffizienz findet sich ein Hypokortisolismus mit einem (inadäquat) niedrigen ACTH.

Diagnostischer Goldstandard zum Ausschluss eines Hyperkortisolismus ist der niedrig dosierte Dexamethasonsuppressionstest (1 mg über Nacht). Als sicherer Ausschluss eines Hyperkortisolismus wird die Suppression des Serumkortisols nach 1 mg Dexamethason auf < 1,8 µg/dl angesehen. Ein Hyperkortisolismus ist aber vor allem auch eine klinische Diagnose, weshalb eine ausführliche körperliche Untersuchung erforderlich ist, um klinische Zeichen eines Cushing-Syndroms zu erkennen. Allerdings kann die Diagnostik eines Hyperkortisolismus bei schwer kranken Patient*innen sehr schwierig sein, weil situativ bedingt hohe Serumkortisolkonzentrationen auftreten können. So ist im Fall eines positiven Erstliniensuppressionstest mit 1 mg Dexamethason ein zusätzlicher hoch dosierter Dexamethasonsuppressionstest mit 8 mg sinnvoll. Für die Interpretation dieses Tests und für die weitere Diagnostik ist jedoch ebenfalls endokrinologische Expertise erforderlich.

In der Mehrzahl der Fälle ist ein ACTH-unabhängiger Hyperkortisolismus auf eine ACS zurückzuführen, wobei der Hyperkortisolismus mild ist und die Patient*innen keine typischen klinischen Merkmale eines Hyperkortisolismus aufweisen. Für eine schnelle Stratifizierung in AC und ACS ist daher neben den entsprechenden Labortests (basales ACTH, Kortisol sowie Dehydroepiandrosteronsulfat [DHEAS] zusammen mit der Ausscheidung von freiem Kortisol im 24 h-Urin) der klinische Aspekt mit dem Vorhandensein von klinischen cushingoiden Veränderungen entscheidend. Ein niedriger DHEAS-Spiegel wurde bereits als vielversprechender diagnostischer Biomarker für die ACS postuliert [16, 21]. Darüber hinaus sind weitere laborchemische Entwicklungen zur Unterscheidung von Patient*innen mit ACS und AC im Gange, unter anderem die Entwicklung von Steroidpanels auf Basis der Flüssigchromatographie-Tandem-Massenspektrometrie (LC-MS/MS). Diese Methoden sind allerdings in der Routinediagnostik nicht verfügbar [27]. Sowohl bei AC als auch bei ACS sollte jedoch eine Bildgebung mit der Frage nach einer Nebennierenraumforderung oder Hyperplasie durchgeführt werden. Bei einem bestätigten ACTH-abhängigen Cushing-Syndrom sollten zusätzliche diagnostische Untersuchungen erfolgen, unter anderem ein hoch dosierter Dexamethasontest, Kortikotropin-Releasing-Hormon(CRH)-Test sowie bildgebende Verfahren und Sinus-petrosus-Katheter zur Unterscheidung zwischen einem Morbus Cushing (ACTH-produzierendes Hypophysenadenom) und einer ektopen ACTH-Quelle [16]. Diese Aspekte werden hier nicht weiter betrachtet.

### Therapeutisches Management

Unabhängig davon, ob die Patient*innen spontan atmen können, mit kontinuierlichem positivem Atemwegsdruck (CPAP) oder maschinell invasiv über einen Tubus beatmet werden müssen, sollten Patient*innen mit schwerer SARS-CoV-2-Infektion und dringendem Verdacht auf oder neu diagnostizierter PAI unverzüglich eine Kurzinfusion von 100 mg Hydrokortison i.v. erhalten und auf eine hohe Stressdosis Hydrokortison, das heißt 200 mg über 24 h, eingestellt werden. Alternativ können 50 mg Hydrokortison als Bolusinjektion i.v. oder i.m. alle 6 h verabreicht werden. Parallel ist eine ausreichende intravenöse Flüssigkeitssubstitution zur hämodynamischen Stabilisierung mit isotonischer Kochsalzlösung erforderlich. Kinder mit PAI sollten unverzüglich eine parenterale Injektion von 50 bis 100 mg/m^2^ Hydrokortison erhalten. Anschließend sollte eine parenterale Applikation von 50 bis 100 mg Hydrokortison/24 h erfolgen, vorzugsweise aufgeteilt in 4 parenterale Dosen alle 6 h. Bei erwachsenen Patient*innen mit einer bekannten PAI kann eine zuvor bestehende Therapie mit Fludrokortison solange pausiert werden, wie eine intravenöse Steuerung des Flüssigkeits- und Elektrolythaushalts über eine Infusionstherapie erfolgt. Bei Patient*innen mit einer PAI, die bei einer Infektion mit SARS-CoV‑2 ein „acute respiratory distress syndrome“ (ARDS) entwickeln, sollte die Glukokortikoidtherapie mit einer maximalen Dosierung (200 mg/24 h über Perfusor) fortgesetzt werden.

Diese Maßnahmen sollten durchgeführt werden, bis eine hämodynamische Stabilisierung eingetreten, keine mechanische Beatmung mehr erforderlich und eine deutliche klinische Verbesserung zu verzeichnen ist. Danach kann die Glukokortikoidersatzdosis entsprechend der weiteren klinischen Verbesserung schrittweise reduziert werden. Zum Zeitpunkt der Entlassung sollte die Dosis auf das Doppelte der ursprünglichen Ersatzdosis reduziert sein. Wurde vor der intensivmedizinischen Therapie Fludrokortison verabreicht, sollte es bei Erwachsenen wieder in der üblichen Dosis verabreicht werden, wenn die Gesamttagesdosis von Hydrokortison < 50 mg beträgt [24, 26].

Operationen sollten bei SARS-CoV-2-Infektion und Hyperkortisolismus möglichst verschoben werden

Patient*innen mit schwerer SARS-CoV-2-Infektion und neu diagnostiziertem Hyperkortisolismus sollten sehr engmaschig klinisch überwacht werden, da sie immunkompromittiert sind und möglicherweise weniger ausgeprägte entzündliche oder fieberhafte Reaktionen zeigen. Das klinische Monitoring sollte sich auf Symptome und Anzeichen wie Dyspnoe, Husten, Dysgeusie, Anosmie und Diarrhö stützen. Bei Patient*innen mit ausgeprägtem Hyperkortisolismus ist von einem erhöhten Risiko für eine länger andauernde Virusinfektion sowie für die Entwicklung opportunistischer Infektionen auszugehen, insbesondere in Bezug auf atypische bakterielle, *Pneumocystis jirovecii*- und invasive Pilzinfektionen. Auch das Risiko einer Sepsis und die Mortalität sind als erhöht einzustufen. Daher sollten im Fall einer SARS-CoV-2-Infektion bei Patient*innen mit Hyperkortisolismus eine verlängerte antivirale Behandlung, eine *Pneumocystis*-Prophylaxe und eine empirische Prophylaxe mit Breitbandantibiotika in Betracht gezogen werden. Sowohl ein Cushing-Syndrom als auch eine Infektion mit SARS-CoV‑2 ist mit einer Hyperkoagulabilität assoziiert, sodass bei Patient*innen mit einem Hyperkortisolismus von einem erhöhten thromboembolischen Risiko auszugehen ist. Es wurde gezeigt, dass eine Therapie mit niedermolekularem Heparin bei Patient*innen mit SARS-CoV-2-Infektion die thromboembolische Komplikationsrate und Mortalität senkt, sodass diese Antikoagulation insbesondere auch bei SARS-CoV-2-infizierten Patient*innen mit einem Hyperkortisolismus dringend empfohlen wird. Operationen sollten bei Patient*innen mit SARS-CoV-2-Infektion und Hyperkortisolismus möglichst verschoben werden, um die Folgen einer postoperativen Immunsuppression und thromboembolische Risiken zu vermindern. Gegebenenfalls kann bei einem schweren endogenen Hyperkortisolismus in solchen Fällen während der SARS-CoV-2-Pandemie vorübergehend eine kortisolsenkende medikamentöse Therapie, etwa mit Ketoconazol, Metyrapon, Osilodrostat oder Etomidat, erwogen werden [28].

Antidiabetische Therapie sollte kritisch hinsichtlich sogenannter „sick day rules“ überprüft werden

Im Zusammenhang mit der beim Hyperkortisolismus häufig zu beobachtenden gestörten glykämischen Kontrolle („Steroiddiabetes“) verweisen wir auf die neuen praktischen Empfehlungen der Deutschen Diabetes Gesellschaft zum Diabetesmanagement bei Patient*innen mit einer „coronavirus disease 2019“(COVID-19). Infizierte Patient*innen ohne vorbekannten Diabetes mellitus sollten durch Glukosemonitoring oder Hämoglobin‑A_1c_(HbA_1c_)-Bestimmung bezüglich einer Neumanifestation getestet werden. Bei Diabetes und schweren Verläufen von SARS-CoV-2-Infektionen, insbesondere solchen mit Krankenhausaufenthalt, kann initial Insulin eingesetzt werden, da hier am wenigsten Komplikationen (beispielsweise Laktatazidose, Ketoazidose) und Medikamenteninteraktionen (unter anderem auch mit experimentellen antiviralen Therapeutika wie Hydroxychloroquin, Lopinavir/Ritonavir oder Remdesivir) zu erwarten sind. Es wird empfohlen, eine antihyperglykämische Therapie bei Blutglukosewerten (nüchtern) ab 180 mg/dl (10 mmol/l) zu beginnen bzw. zu intensivieren. Bei Werten unter 110 mg/dl (6,1 mmol/l) sollte hingegen eine antidiabetische Therapie pausiert bzw. angepasst werden, gegebenenfalls sollten sogar blutglukoseerhöhende Maßnahmen ergriffen werden. Die antidiabetische Therapie sollte kritisch hinsichtlich sogenannter „sick day rules“ überprüft und angepasst werden. Dies gilt insbesondere für Natrium-Glukose-Kotransporter-2(SGLT-2)-Inhibitoren zur Vermeidung einer atypischen Ketoazidose und für Metformin hinsichtlich einer Laktatazidose. Sulfonylharnstoffe sollten wegen der Gefahr von Hypoglykämien infolge einer Kumulation bei (transienter) Niereninsuffizienz pausiert werden.

**Figure 3:**
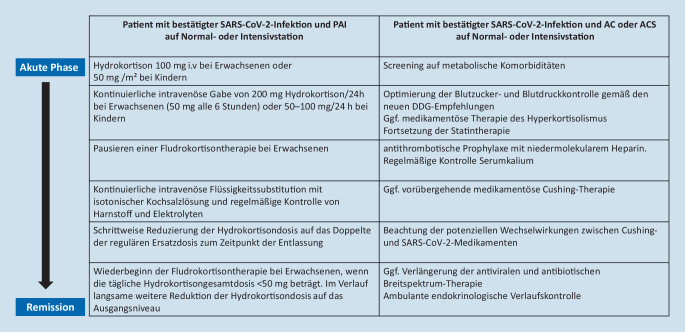

Der Zielblutdruck beträgt < 135/80 mmHg unter antihypertensiver Therapie. Ein bestehendes antihypertensives Behandlungsregime einschließlich Angiotensin-converting-enzyme(ACE)-Hemmer und Angiotensinrezeptorblocker sollte zunächst möglichst beibehalten werden. Eine gegebenenfalls bestehende Therapie mit Statinen sollte fortgesetzt werden. Lediglich bei einem Kreatinkinaseanstieg sollte das Statin pausiert werden. Beim Einsatz einer antiviralen Begleitmedikation, beispielsweise mit Hydroxychloroquin, Lopinavir/Ritonavir, Remdesivir oder Tocilizumab, sollten Medikamenteninteraktionen berücksichtigt werden, insbesondere mit antidiabetischen Substanzen [29, 30]. In Abb. 3 ist das Management von Patient*innen mit SARS-CoV-2-Infektion und Funktionsstörungen der Nebennierenrinde dargestellt.

## Fazit für die Praxis

- Bei Patient*innen mit einer Severe-acute-respiratory-syndrome-coronavirus-2(SARS-CoV-2)-Infektion sollten Funktionsstörungen der Nebennierenrinde – insbesondere eine primäre Nebennierenrindeninsuffizienz und ein Cushing-Syndrom – als Begleiterkrankungen bereits in der akuten Phase erkannt bzw. ausgeschlossen werden.
- Bei diesen Erkrankungen ist mit vermehrten Komplikationen und einer erhöhten Mortalität im Falle einer SARS-CoV-2-Infektion zu rechnen.
- Im Initialstadium der SARS-CoV-2-Erkrankung erleichtert das rechtzeitige Erkennen die Therapie und verbessert die Prognose.
